# Quorum Sensing and Mobility Inhibition of Pathogenic Bacteria by *Fulvifomes mexicanus* sp. nov.

**DOI:** 10.3390/molecules30112278

**Published:** 2025-05-22

**Authors:** Angelica Bolaños-Nuñez, Michelle Martínez-Pineda, Ricardo Valenzuela, Mario Figueroa, Albert D. Patiño, Everardo Curiel-Quesada, César Ramiro Martínez-Gonzáles, Rodrigo Villanueva-Silva, Tania Raymundo, Abigail Pérez-Valdespino

**Affiliations:** 1 Departamento de Botánica, Escuela Nacional de Ciencias Biológicas, Instituto Politécnico Nacional, Prolongación de Carpio y Plan de Ayala S/N, Col. Santo Tomás, Mexico City 11340, CDMX, Mexico; angelicabolanosn@gmail.com (A.B.-N.); mmartinezpin@ipn.mx (M.M.-P.); rvalenzg@ipn.mx (R.V.); 2 Departamento de Bioquímica, Escuela Nacional de Ciencias Biológicas, Instituto Politécnico Nacional, Prolongación de Carpio y Plan de Ayala S/N, Col. Santo Tomás, Mexico City 11340, CDMX, Mexico; ecqmixcoaqdf@gmail.com; 3 Departmento de Farmacia, Facultad de Química, Universidad Nacional Autónoma de México, Mexico City 04510, CDMX, Mexico; mafiguer@unam.mx (M.F.); albert.patino@comunidad.unam.mx (A.D.P.); 4 Herbario Micológico José Castillo Tovar, Instituto Tecnológico de Ciudad Victoria, Tecnológico Nacional de México, Boulevard Emilio Portes Gil No. 1301, Ciudad Victoria 87010, Tamaulipas, Mexico; cesar.ramiro.mg@gmail.com; 5 Programa de Investigadoras e Investigadores por México de la Secretaría de Ciencia, Humanidades, Tecnología e Innovación, Departamento de Toxicología, Centro de Investigaciones y Estudios Avanzados del IPN, Av. Instituto Politécnico Nacional 2508, Col. San Pedro Zacatenco, Mexico City 07360, CDMX, Mexico; rodrigo.villanueva@cinvestav.mx

**Keywords:** *Fulvifomes mexicanus* sp. nov., metabolic analysis, antipathogenic activity

## Abstract

The development of antimicrobial resistance drives the search for molecules capable of inhibiting bacterial virulence. Fungi of the Basidiomycota phylum constitute an important source of compounds with antimicrobial activity. The present paper describes a new species named *Fulvifomes mexicanus* sp. nov. based on morphological and phylogenetic analyses. The methanolic extract of basidiome of this fungus inhibited the motility of *Pseudomonas aeruginosa* ATCC 9027 and the production of violacein by *Chromobacterium violaceu*m CV026. The metabolomic study of the extract by liquid chromatography–high-resolution electrospray ionization mass spectrometry (LC-HRESIMS) and molecular networking analyses revealed the presence of a complex composition of metabolites including hispidin derivatives, terpenoids, phenols, furanones, alkylglycerols, pyrones, and γ-butyrolactones, among others. Overall, this work represents the first chemical and biological study of a new species of *Fulvifomes mexicanus* as a source of antipathogenic metabolites for the development of novel antimicrobial agents.

## 1. Introduction

The increasing prevalence of pathogenic bacteria resistant to commercially available drugs and the limited range of novel effective therapeutic agents represent a significant challenge for global health services [1]. In this context, it is crucial to identify novel compounds with antibiotic properties or substances that inhibit the expression of virulence genes in resistant bacteria and enable the host immune system to combat the infection effectively [2,3,4,5,6,7]. In many pathogenic bacteria, the expression of virulence factors is mainly controlled by Quorum Sensing (QS). QS is a communication system between bacteria (fluctuations in the cell population) that determines when the right time is for particular genes to be expressed [8]. QS inhibitors are used to block these virulence processes and different bacterial species like *Pseudomonas aeruginosa* and *Chromobacterium violaceum*, with QS-based factors such as exopolysaccharide production, swimming and swarming motility, and the inhibition of violacein production, are used as a models to target bacterial cell-to-cell communication [9].

Fungi are considered important reservoirs of compounds that favor their capacity to grow under different environments and hostile conditions by inhibiting the microbial growth of other organisms [10]. Therefore, the characterization of fungal secondary metabolites with antipathogenic activity is a tool to counteract multidrug resistance [11]. Moreover, these metabolites can also become sources of structural prototypes for chemical modification to prolong the time span of the compound in question in the face of bacterial adaptability [12].

Fungi of the genus *Fulvifomes* (*Hymenochaetaceae*) are xylophagous basidiomycetes that cause white rot in trees in tropical deciduous forests, tropical evergreen forests, xerophytic scrublands, and mangroves [13,14,15]. *Fulvifomes* spp. are characterized by being annual or perennial, pileate, and effusible-to-sub-stipitate; having corky to woody consistency, homogeneous or duplex contexts, hymenophores with circular or angular pores and entire margins, monomitic or dimitic hyphal systems, and the absence of setae; and having subglobose-to-ellipsoidal, yellowish-to-brownish, slightly thick-walled to thick-walled basidiospores [16,17,18]. There have been few reports on the cytotoxic activity of secondary metabolites of *Fulvifomes* spp. [19,20]. However, there have been no reports on the antibacterial or antipathogenic activity of fungi of this genus. Therefore, this work established the metabolomic profile and antipathogenic capacity of a *Fulvifomes* specimen isolated from the Sierra de Alamos Natural Protected Area in Sonora, Mexico.

## 2. Results

### 2.1. Taxonomy

#### Basidiomycota, Agaricomycetes, Hymenochaetales, Hymenochaetaceae

**Figure 1:**
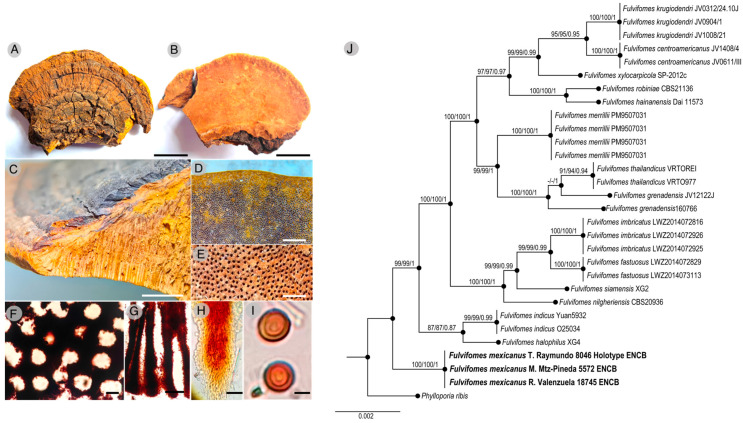
Basidiomata of the type specimens examined (**A**–**I**) and phylogenetic reconstruction based on the ITS and LSU regions (**J**). MP and BI analyses recovered identical topologies concerning the relationships among the main clades of *Fulvifomes* members. For each node, the following values have been provided: MP (≥70%, left)/ML bootstrap (≥70%, middle) and the BI posterior probability (≥0.85, right). The scale bar represents the expected number of nucleotide substitutions per site.

*Fulvifomes mexicanus* Mart.-Pineda, R. Valenz. & Raymundo sp. nov.
Figure 1: A–I
Mycobank: 859269
Type. Mexico. Sonora State, Alamos-Río Cuchujaqui Biosphere Reserve, Alamos Municipality, Promontorios, LN 26°59′55″, LW 109°03′21″, alt. 370 m, 26 October 2018, Tropical dry forest, T. Raymundo 8046 (Holotype ENCB).
Etymology. mexicanus (Lat.): referring to country of the locality type.
Description. Basidiomata 70–135 × 50−70 × 25–55 mm, perennial, pileate-sessile, aplanate with umbo, broadly attached to the substrate, consistency woody hard. Pileus brown (7D-E8) to reddish brown (8E7) with tones dark brown (8F8,4) and the old part of the pileus (center) greyish brown (8F3) to black, concentrically zonate to sulcate, with some lines brownish grey (7E2) to the margin, slightly velvety in younger zones, glabrous, to slightly rimose to cracked, with a distinct crust, up to 5 mm thick to the center. Margin obtuse, sterile to fertile, yellowish brown (5E8), brown (6E8) to dark brown (7F6). Hymenophore poroid, brown (7D7) to dark brown, pores circular, 5–7 per mm, dissepiments (70–) 85–90 (–100) µm thick, and lumen 140–160 µm diam. Tubes brown (7E8), woody hard, up to 45 mm thick, tube layers distinctly stratified with intermittent context layers, individual tube layer up to 3 mm thick. Context up to 10 mm thick, brown (6E8) to dark brown in the surface.

Hyphal system dimitic, tramal hyphae slightly interwoven, with generative hyphae simple septate, hyaline to yellowish in KOH 5%, some light brown, simple to branched, thin walled, 3–5 µm in diam.; skeletal hyphae aseptate, yellowish brown to reddish brown in KOH 5%, unbranched, thick walled, with distinct lumen, 3–6 µm in diam.; contextual hyphae subparallel to slightly interwoven, with generative hyphae simple septate, hyaline, yellowish to light brown in KOH 5%, simple to branched, thin walled, 3–5 µm in diam.; skeletal hyphae dominant, aseptate, yellowish brown to reddish brown in KOH 5%, unbranched, thick walled, with distinct lumen, 4–7 µm in diam. Setae absent in the basidiome. Basidia not observed. Basidiospores 5–6 (−7) × 4–5 µm (average: 5.5 × 4.5, n = 30/1), globose to subglobose in frontal view, broadly ellipsoid to ellipsoid with a plane side in lateral view, (Q = 1–1.15 in frontal view, 1.2–1.5 in lateral view), yellowish brown to reddish Brown in KOH 5%, smooth, thick walled. Habitat. This species grows on live wood of Lysiloma divaricatum (Jacq.) J.F. Macbr. in tropical dry forest and causes a white rot. 

Additional specimens examined. Mexico. Sonora State, Alamos-Río Cuchujaqui Biosphere Reserve, Alamos Municipality, Promontorios, LN 26°59′55″, LW 109°03′21″, alt. 370 m, 26 October 2018, Tropical dry forest, M. Martínez-Pineda 5572 (paratype ENCB); R. Valenzuela 18745 (paratype: ENCB).

Remarks. This species is different of the others species of Fulvifomes by its aplanate with umbo, glabrous to cracked and a distinct crust in the basidiomata, its hymenophore with circular pores, 5–7 per mm, its globose basidiospores in frontal view and broadly ellipsoid to ellipsoid with a plane side in lateral view and growing on live wood of Lysiloma divaricatum in tropical dry forest in Mexico. 

### 2.2. Phylogenetic Analysis of Fulvifomes mexicanus sp. nov.

The concatenated alignment of the internal transcribed spacer (nrITS) and large subunit (LSU) of the nuclear ribosomal DNA comprised 29 taxa with 1314 characters including gaps. The three phylogenetic analyses, maximum parsimony (MP), maximum likelihood (ML), and Bayesian Inference (BI) of the dataset recovered similar topologies (Figure 1J). No significant conflict (bootstrap value > 80%) was detected among the topologies obtained by the different analyses. The MP analysis of the alignment found 958 trees of 141 steps (CI = 0.1001, HI = 0.1007, RI = 0.2561, RC = 0.1830). The best ML tree with a final likelihood value of –18230.008340 was presented. The matrix had 852 distinct alignment patterns, with 3.05% undetermined characters or gaps. Estimated base frequencies were as follows: A = 0.100831, C = 0.101834, G = 0.183504, and T = 0.186420; substitution rates: AC = 1.000814, AG = 1.000972, AT = 1.008648, CG = 1.903712, CT = 4.620044, and GT = 1.1000000; the gamma distribution shape parameter α = 0.000265. The BI analysis showed a standard deviation between the chains stabilized at 0.001 after 3.5 million generations. No significant changes in tree topology configuration or cumulative split frequencies of selected nodes were observed after about 0.35 million generations, which were discarded as 25% burn-in. The analysis produced a phylogenetic tree where *Fulvifomes mexicanus* was shown as a monophyletic group (BS = 100%, BS = 100%, BI *p* = 1). Sequences are available in GenBank under the accession numbers PV522025-PV522027 (ITS) and PV563098-PV563100 (LSU).

### 2.3. Antimicrobial Activity and Inhibition of Swimming Motility

The methanolic extract of *Fulvifomes mexicanus* sp. nov. showed antibacterial activity against *Staphylococcus aureus* and *Vibrio parahaemolyticus* with a minimum inhibitory concentration (MIC) of 2.3 mg/mL and *Chromobacterium violaceum* with an MIC of 4.7 mg/mL. The least susceptible bacteria to this extract were the pathogens *Salmonella typhimurium* and *Pseudomonas aeruginosa* with MIC values of 9.4 and 18.8 mg/mL, respectively. No activity was observed for the chloroform extract of the fungus.

**Figure 2:**
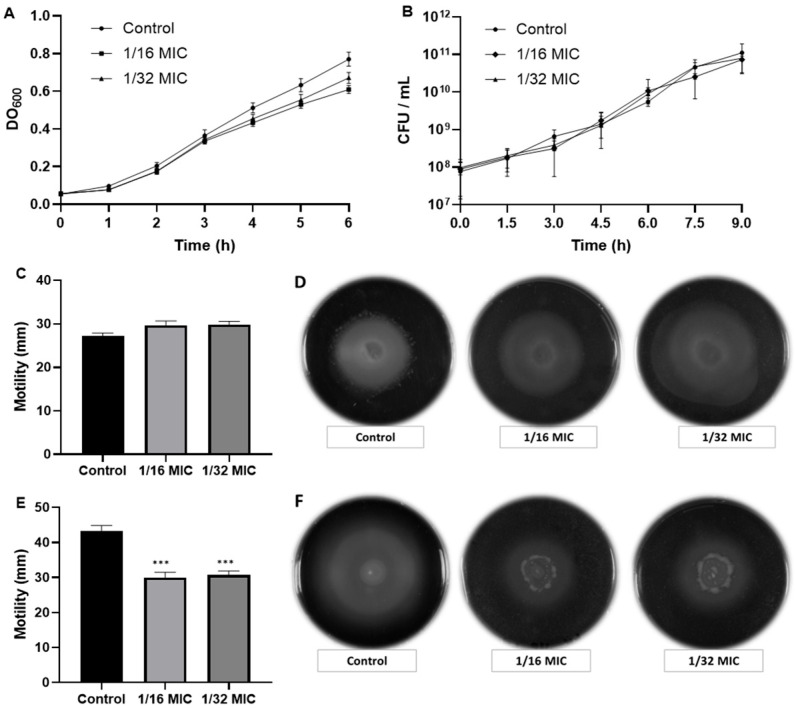
Effect of *F. mexicanus* sp. nov. methanolic extract on bacterial motility. Panels (**A**,**B**) show the growth curves of *S. typhimurium* ATCC 7251 and *P. aeruginosa* ATCC 9027, respectively. Panels (**C**,**D**) show the motility assay in *S. typhimurium* ATCC 7251, and (**E**,**F**) of *P. aeruginosa* ATCC 9027. We noted significant difference from control generated by Dunnet’s test, *** *p* < 0.0001.

Based on the latter, the impact on swimming-type bacterial motility was established only for the fungus’s methanolic extract against *S. typhimurium* ATCC 7251 and *P. aeruginosa* ATCC 9027 at concentrations of 1/16 and 1/32 MIC. Interestingly, the methanolic extract inhibited the motility of *P. aeruginosa* by approximately 30% while no effect was observed on the motility of *S. typhimurium* (Figure 2). These data are important because the anti-motility compounds were aimed at specific targets as the mobility between the two types of bacteria was different.

### 2.4. Inhibition of Violacein Production

**Figure 3:**
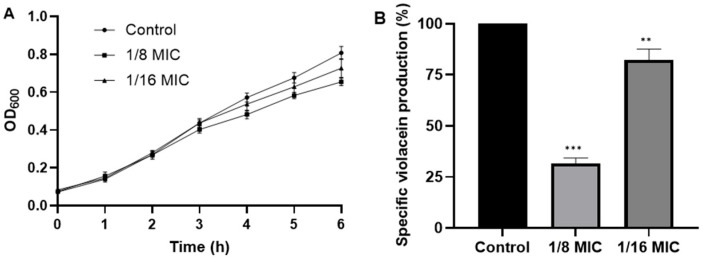
(**A**) Effect of *F. mexicanus* sp. nov. methanolic extract on growth and (**B**) specific violacein production of *C. violaceum* CV026. Significant difference with respect to control was noted (Dunnet’s test), *** *p* < 0.0001; ** *p* < 0.01.

The effect of the methanolic extract of *F. mexicanus* sp. nov. on the Quorum sensing (QS) of *C. violaceum* was evaluated by reducing the production of violacein at sub-MIC concentrations. The results showed that the extract reduced the violacein concentrations at 1/8 and 1/16 of the MIC by 68.6% and 17.8%, respectively (Figure 3).

### 2.5. Metabolomic Profiling and Molecular Networking

**Figure 4:**
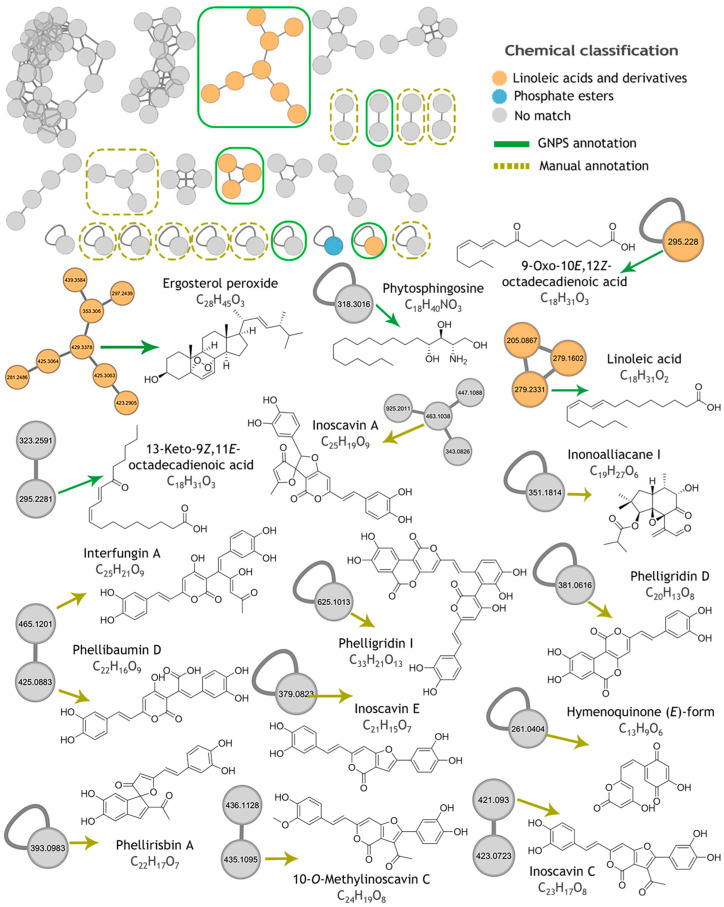
Molecular networking analysis of *F. mexicanus* sp. nov. methanolic extracts with automatic (by GNPS) and manual annotations. Molecular formulas correspond to the observed adduct.

The untargeted metabolomic analysis of the fungal methanolic extract was performed using LC-HRESIMS-MS/MS data and the global natural product social molecular networking (GNPS) platform. Metabolites in the extract were grouped into two-hundred-and-ninety-eight nodes, forming 12 clusters with at least three nodes, 12 with two nodes, and 194 singletons. From this, 4% of the nodes were associated with linoleic acid and derivatives, 0.4% were identified as phosphate esters, and 95% were not assigned to any chemical subclass (Figure 4 and Table 1). To expand the library of metabolites annotated by molecular networking, accurate mass values, MS/MS fragment ions, and UV data were used to identify metabolites manually by comparison with the literature data and information available at the Dictionary of Natural Products (https://dnp.chemnetbase.com, accessed on 28 February 2025). To perform the presumptive identification, those from the *Hymenochaetaceae* family with a reported MS2 spectrum, at least four ions matching, and similar fragmentation patterns from predictor CFM-ID 4.0 were used as the reference. From this analysis, 11 additional metabolites were annotated, including a pyrone, a sesquiterpenoid, and hispidine derivates (Table 2).

## 3. Discussion

Bacterial infection control has been limited by the emergence of multidrug-resistant strains [1,21]. An alternative to counteract this problem is to identify and isolate compounds that attenuate the virulence of pathogenic microorganisms [22]. The disruption of Quorum sensing (QS) and bacterial motility is proposed as a way to fight infection by modulating bacterial pathogenicity and weakening the pathogen against the immune system [23].

Fungi of the *Hymenochaetaceae* family have been used in traditional medicine to treat, among others, several types of infections [24,25]. *Fulvifomes mexicaus* sp. nov. is a species characterized by the formation of ungulate basidiomata with reddish-brown spores growing on living trees of *Lysiloma latisiquum.* These characteristics, together with phylogenetic analysis, clearly distinguish it from other species of the genus. This work has shown the methanolic extract’s biological activity and metabolomic profile from the *F. mexicanus* sp. nov. basidiome. The methanolic extract reduced the motility of *P. aeruginosa* ATCC 9027. This bacterium exhibits a flagellum-dependent swimming motility, which plays an important role in its movement during the infection process, as well as in the formation of biofilm, which contributes to antibiotic resistance [26,27,28,29,30,31,32]. The extract did not restrict the swimming motility of *S. typhimurium* ATCC 7251, which is relevant since anti-motility compounds aim at specific targets. The decrease in motility in *P*. *aeruginosa* ATCC 9027 could have been related to the presence of ergosterol peroxide, a terpenoid compound associated with the inhibition of the assembly of motility structures due to its interaction with the bacterial membrane and cytoplasm [33]. A wide range of biological actions have also been attributed to this compound, including antioxidant, antimicrobial, antiparasitic, antiviral, antifungal, cytotoxic and immunomodulatory activities [34,35,36,37,38,39].

QS regulates the expression of virulence genes, and its inhibition is therefore considered an alternative to antibiotics to avoid the development of bacterial resistance [40,41]. The methanolic extract inhibited violacein production by up to 70%. Previous reports showed that basidiomycetes of the genus *Phellinus* (*Hymenochaetaceae*) reduced violacein production by *C. violaceum* CV026 [42,43]. The reduction in violacein production is mainly caused by analogs of autoinducers, phenols, flavonoids, furanones, terpenoids, alkylglycerols, pyrones, and γ-butyrolactones with AHL-like structures or their reactive groups capable of interacting with the target site, which have been associated with the inhibition of pigment synthesis [44,45,46,47,48,49,50]. Many of these compounds were identified in the metabolic profile of *F. mexicanus* sp. nov. It is imperative to acknowledge that the biological activities were evaluated using crude extracts. Consequently, the presence of combined or pure compounds may have contributed to the observed biological activities. On the other hand, if the observed effect on QS was due to a single component in the extract, its activity in pure form would probably have been much higher, making very interesting its isolation and characterization.

The metabolomic profile of species belonging to the genus *Phellinus sensu lato*, from which the genus *Fulvifomes* was segregated, has been described by several authors [51]. These species concur on the presence of alkaloids, tannins, flavonoids, steroids, glycosides, and phenolic compounds, which are associated with a variety of biological activities [52,53,54]. For example, the methanolic extracts of *Fuscoporia ferruginosa*, *Inonotus hispidus*, and *Phenillus* spp. contain polyphenols with antioxidant activity and could also be responsible for their antibacterial activity [55,56,57]. Nevertheless, this was the first study in which the identification and characterization of the compounds of the *Fulvifomes* genus was performed.

The *Fulvifomes mexicanus* sp. nov. methanolic extract revealed the presence of various hispidin-derived compounds such as interfungin A, 10-O-Methylinoscavin C, phellibaumin D, phelligridin I, and inoscavin types A, C, and E, which have shown a range of biological activities, including antioxidant, antibacterial, antitumoral, cytotoxic, and antiviral activities. The generation of these compounds is facilitated by the oxidative coupling of hispidin with additional phenolic compounds or other hispidins, a process catalyzed by peroxidases [58,59,60]. Lipid compounds, such as linoleic acid, a metabolite present in cell membranes, were also abundant in the extract. The lipid derivatives have been implicated in the growth and development of the fruiting bodies of various fungi [61,62]. Additionally, clusters of unidentified metabolites, devoid of any chemical classification, suggests their potential as subjects for further study, with the possibility of yielding novel chemical entities.

## 4. Materials and Methods

### 4.1. Fungal Specimen

The specimen was collected in the Sierra de Alamos Natural Protected Area, Sonora, Mexico and preserved at the Herbarium of the National School of Biological Sciences of the National Polytechnic Institute, with access code TR5566. The identification and characterization of the fungus was carried out according to the guidelines of Dai [63] and Gilbertson and Ryvarden [64]. The size, shape, color, consistency, texture of the basidiome, and the shapes and sizes of the pores were analyzed. The Kornerup and Wanscher [65] table was used to establish the colors of the structures. Sections were made to describe micromorphological characters, including spores, basidia, cystidia, hyphal system, and hymenophores [66]. The species was confirmed by sequence analysis of the internal transcribed spacer of the nuclear ribosomal DNA (nrITS) [67].

### 4.2. Genetic Identification

Genomic DNA was extracted from 50–100 mg of the basidiome using the CTAB method [68]. The process of the internal transcribed spacer of the nuclear ribosomal DNA (nrITS) and large subunit (LSU) amplification were carried out [69]. The PCR products were purified with the ExoSAP Purification kit (Affymetrix, Cleveland, OH, USA) following the manufacturer’s instructions. Amplicons were quantified and sequenced with an Applied Biosystem model 3730XL (Applied BioSystems, Foster City, CA, USA). Subsequently, the sequences were analyzed, edited, and assembled using the BioEdit v7.0.1 [70] to generate a consensus sequence.

In order to study phylogenetic relationships, the consensus sequences were compared with those deposited in the GenBank of the National Center for Biotechnology Information (NCBI) using the tool BLAST v2.2.19 [71]. The ITS and LSU regions were aligned using the online version of MAFFT v7 [72]. The alignments were revised with PhyDE v0.9971 [73] followed by minor manual adjustments to ensure character homology between taxa. The data were analyzed using maximum parsimony (MP), maximum likelihood (ML), and Bayesian Inference (BI). MP analysis was carried out in PAUP* 4.0b10 [74] using the heuristic search mode, 1000 random starting replicates, and TBR branch swapping, with MULTREES and Collapse on. Bootstrap values were estimated using 1000 replicates under the heuristic search mode, each with 100 random starting replicates. ML analysis was carried out in IQ-tree v1.6.12 [75] with a GTR + G model of nucleotide substitution. To assess branch support, 1000 rapid bootstrap replicates were run with the GTRGAMMA model. BI was carried out in MrBayes v3.2.7 [76] with four chains and the best evolutionary model for alignment was sought using Partition Finder [77]. The matrix included two simultaneous runs of Montecarlo chains, with temperature set to 0.2 and sampling 10 million generations (standard deviation ≤ 0.1). Chain convergence was visualized with TRACER v1.7.1 [78]. The remaining trees were used to calculate a 50% majority-rule consensus topology and posterior probabilities (PP). Trees were visualized and optimized in FigTree v1.4.4 [79].

### 4.3. Extracts Preparation

The fungus’s fruiting bodies were washed with distilled water, dried at 42 °C, and ground into powder. Thirty grams of the powder were mixed with 300 mL of methanol or chloroform (Meyer, CDMX, Mexico). Each mixture was incubated at room temperature for a period of four days, shaking constantly. Subsequently, each mixture was filtered and concentrated under reduced pressure. Dried extracts were dissolved in hexane (Meyer, CDMX, Mexico), evaporated to a completely dry solid, and kept at 4 °C until use.

### 4.4. Antimicrobial Activity and Minimum Inhibitory Concentration (MIC).

The extracts were tested for antibacterial activity against *Salmonella typhimurium* ATCC 7251, *Pseudomonas aeruginosa* ATCC 9027, *Vibrio parahaemolyticus* (clinical isolate), and *Staphylococcus aureus* (clinical isolate). All pathogens were cultured in Mueller–Hinton (MH) broth (Difco, Sparks, MD, USA) for 24 h at 37 °C, adjusted to 0.5 on the McFarland scale, and spread onto MH agar plates using a sterile swab. After drying, 10 µL of each fungal extract (37.5 mg of extract in 1:1 DMSO-H2O) was dropped onto the plate, on a sterile filter paper disk. Chloramphenicol (Sigma-Aldrich, St. Louis, MO, USA) disks (30 µg/mL) was used as antibacterial activity control and 1:1 DMSO-H2O as solvent control. Plates were incubated for 24 h at 37 °C. Assays were performed in triplicate [80,81]. In addition, the minimum inhibitory concentration (MIC) of the fungal extracts was established using the broth microdilution method in 96-well microtiter plates, following the protocol of the Clinical and Laboratory Standards Institute (CLSI) [82].

### 4.5. Mobility Inhibition Tests

*Salmonella typhimurium* ATCC 7251 and *P. aeruginosa* ATCC 9027 were used to perform swimming motility assays. The growth curves for each pathogen were determined in the presence of extracts diluted to 1/4, 1/8, 1/16, and 1/32 of the MIC. Semi-solid Luria Broth (LB) (0.2% agar) medium plates supplemented with the extracts were used for the motility assay. Plates were inoculated with 5 µL of culture adjusted to 0.1 optical density at 600 nm (OD_600_). The radial growth diameter was measured after incubation of the plates at 30 °C for 36 h [83].

### 4.6. Inhibition of Violacein Production.

*Chromobacterium violaceum* CV026 was used to evaluate the effect of the extracts on QS [84]. First, the growth curve was established with the extract diluted to 1/8, 1/16, and 1/32 of the MIC. The strain CV026 was inoculated in LB medium (Difco, MD, USA) to 0.1 OD_600_. Then, the culture was supplemented with N-hexanoyl-L-homoserine lactone (C6-AHL) (Sigma-Aldrich, St. Louis, MO, USA) at a final concentration of 50 μM. Subsequently, the extracts were diluted until 1/16 and 1/32 of the MIC was reached. DMSO (final concentration 2.4%) and C6-AHL without extracts were used as controls. Tubes were incubated at 32 °C for 16 h with continuous shaking at 200 rpm. Upon completion of the incubation time, cell density was determined by absorbance at 720 nm by using the LB medium as a blank. Finally, the production of violacein was measured. Briefly, 500 μL of the bacterial culture was placed in a 2 mL tube, and then, 500 μL of acetone was added. The tubes were vortexed and centrifuged at 15,000 rpm for 1 min, and the supernatants were separated. The specific production of violacein was calculated by dividing the value of the reading at 577 nm by that at 720 nm. Each experiment was performed 3 times.

### 4.7. LC-MS, Untargeted Metabolomic and Feature-Based Molecular Network Analysis

Extracts (1 mg/mL) were analyzed on an Acquity UPLC (Waters Corp., Milford, MA, USA) coupled to a Q Exactive Plus mass spectrometer (Thermo Fisher Scientific, Waltham, MA, USA). LC analysis was performed on an Acquity BEH C18 column (130 Å, 1.7 µm, 2.1 mm × 50 mm; Waters) at 40 °C, with a gradient system from 15:85 CH_3_CN–0.1% aqueous formic acid to 100% of CH_3_CN in 8 min, then held for 1.5 min with CH_3_CN and returned to the starting conditions. The flow rate was 0.3 mL/min, and the injection volume was 3.0 µL. HRMS-MS/MS data were obtained using an ESI source with positive and negative modes at a full scan range (m/z 150–2000), with the top 5 MSn collected during the whole run. The following settings were used for the MS system: capillary voltage, 5 V; capillary temperature, 300 C; tube lens offset, 35 V; spray voltage, 3.80 kV; sheath and auxiliary gas flow, 30 arbitrary units [85]. Then, MS data were converted to mzXML format using MS Converter tool (ProteoWizard v3.0). A molecular network was created with the Feature-Based Molecular Networking (FBMN) workflow [86] on GNPS (https://gnps.ucsd.edu, accessed on 28 February 2025, [87]). The MS data were first processed with MZmine v3.3.0 [88] and the results were exported to GNPS for FBMN analysis. The spectra in the network were then searched automatically against GNPS spectral libraries [87,89]. The DEREPLICATOR, MS2LDA, and MolNetEnhancer were used to annotate MS/MS-spectrum chemical classification [3,86,90]. The molecular networks were visualized using Cytoscape v3.9.1 [91]. Additionally, manual dereplication was assessed by comparison of HRMS-MS/MS data with those reported in the Dictionary of Natural Products. The manual annotation was made at confidence levels 1 and 2, respectively, according to the metabolomics standards initiative and exact mass accuracy < 5 ppm [92].

### 4.8. Statistical Analysis

GraphPad Prism software v9.5.1 was used to analyze the data using analysis of variance (ANOVA) (α = 0.05). To calculate statistically significant difference values, Dunnett’s test was used for comparisons of means with the control (*p* < 0.05) [93].

## 5. Conclusions

This work represented the first study of the antipathogenic properties of a fungus of the *Fulvifomes* genus. The metabolomic profile of the new species *Fulvifomes mexicanus* sp. nov., which is mainly composed of hispidine derivatives, had not been previously described in this genus and could explain the activity observed in the methanolic extract. Overall, the remarkable chemical diversity detected underscores the potential of *F. mexicanus* as a source of pharmacologically active molecules useful for the development of novel antimicrobial agents.

## Figures and Tables

**Table 1:** Annotation of metabolites in *F. mexicanus* sp. nov. extract by GNPS.

Compound	Observed Ion (*m*/*z*) ^a^	Adduct ^a^	Molecular Formula (Adduct)	Exact Mass	Mass Accuracy (ppm)
13-Keto-9*Z*,11*E*-octadecadienoic acid	295.2281	[M + H]^+^	C_18_H_31_O_3_	295.2268	+4.5
9-Oxo-10*E*,12*Z*-octadecadienoic acid	295.228	[M + H]^+^	C_18_H_31_O_3_	295.2268	+4.2
Linoleic acid	279.2331	[M + H]^+^	C_18_H_31_O_2_	279.2329	+0.5
Phytosphingosine	318.3016	[M + H]^+^	C_18_H_40_NO_3_	318.3003	+4.2
Ergosterol peroxide	429.3378	[M + H]^+^	C_28_H_45_O_3_	429.3363	+3.4

^a^ Values taken from the GNPS platform analysis. ^+^ symbol is not a table footer. It is the positive charge of the ion adduct.

**Table 2:** Manual annotation of metabolites of *F. mexicanus* sp. nov.

Compound	Observed Ion (*m*/*z*)	Adduct	Molecular Formula (Adduct)	Exact Mass	Mass Accuracy (ppm) ^a^	Fungal Source
Interfungin A	465.1201	[M + H]^+^	C_25_H_21_O_9_	465.1180	+4.5	*Hymenochaete xerantica*
Inoscavin A	465.1201	[M + H]^+^	C_25_H_19_O_9_	463.1023	+3.1	*Hymenochaete xerantica*, *Sanghuangporus baumii,* *Fulvifomes fastuosus.*
Phelligridin I (*syn* Inonoblin A)	625.1013	[M + H]^+^	C_33_H_21_O_13_	625.0977	+5.8	*Inonotus obliquus,* *Phellinus igniarius*
10-*O*-Methylinoscavin C	435.1095	[M + H]^+^	C_24_H_19_O_8_	435.1074	+4.7	*Hymenochaete xerantica*
Phelliribsin A	393.0983	[M + H]^+^	C_22_H_17_O_7_	393.0969	+3.6	*Phylloporia ribis*
Hymenoquinone (E)-form	261.0404	[M + H]^+^	C_13_H_9_O_6_	261.0394	+4.0	*Hymenochaete mougeotii*
Phellibaumin D	425.0883	[M + H]^+^	C_22_H_17_O_9_	425.0867	+3.7	*Sanghuangporus baumii*
Inonoalliacane I	351.1814	[M + H]^+^	C_19_H_27_O_6_	351.1802	+3.4	*Inonotus* sp.
Phelligridin D	379.0475	[M + H]^+^	C_20_H_13_O_8_	381.0605	+2.9	*Inonotus obliquus,* *Phellinus igniarius,* *Phellinus linteus*
Inoscavin C	421.0930	[M + H]^+^	C_23_H_17_O_8_	421.0918	+2.9	*Phellinus ellipsoideus,* *Hymenochaete xerantica,* *Phellinus igniarius.*
Inoscavin E	379.0823	[M + H]^+^	C_21_H_15_O_7_	379.0812	+2.8	*Phellinus linteus,* *Hymenochaete xerantica*

^a^ Values obtained from GNPS and LC-HRMS-MS/MS analysis. ^+^ symbol is not a table footer. It is the positive charge of the ion adduct.

## Data Availability

The original contributions presented in this study are included in the article. Further inquiries can be directed to the corresponding author.

## Abbreviations

The following abbreviations have been used in this manuscript:

LC-HRESIMS	Liquid Chromatography–High-Resolution Electrospray Ionization Mass Spectrometry
QS	Quorum Sensing
nrITS	Internal Transcribed Spacer of the nuclear ribosomal DNA
MIC	Minimum Inhibitory Concentration
GNPS	Global Natural Product Social Molecular Networking
MS	Mass Spectrometry
MP	Maximum Parsimony
ML	Maximum Likelihood
BI	Bayesian Inference
MIC	Minimum Inhibitory Concentration
CLSI	Clinical and Laboratory Standards Institute
MH	Mueller–Hinton
LB	Luria Broth
C6-AHL	N-hexanoyl-L-homoserine lactone
FBMN	Feature-Based Molecular Networking
